# ZEB2 controls kidney stromal progenitor differentiation and inhibits abnormal myofibroblast expansion and kidney fibrosis

**DOI:** 10.1172/jci.insight.158418

**Published:** 2023-01-10

**Authors:** Sudhir Kumar, Xueping Fan, Hila Milo Rasouly, Richa Sharma, David J. Salant, Weining Lu

**Affiliations:** Nephrology Section, Department of Medicine, Boston University School of Medicine, Boston Medical Center, Boston, Massachusetts, USA.

**Keywords:** Development, Nephrology, Fibrosis, Mouse models, Pericytes

## Abstract

FOXD1*^+^* cell–derived stromal cells give rise to pericytes and fibroblasts that support the kidney vasculature and interstitium but are also major precursors of myofibroblasts. ZEB2 is a SMAD-interacting transcription factor that is expressed in developing kidney stromal progenitors. Here we show that *Zeb2* is essential for normal FOXD1^+^ stromal progenitor development. Specific conditional knockout of mouse *Zeb2* in FOXD1^+^ stromal progenitors (*Zeb2* cKO) leads to abnormal interstitial stromal cell development, differentiation, and kidney fibrosis. Immunofluorescent staining analyses revealed abnormal expression of interstitial stromal cell markers MEIS1/2/3, CDKN1C, and CSPG4 (NG2) in newborn and 3-week-old *Zeb2*-cKO mouse kidneys. *Zeb2*-deficient FOXD1^+^ stromal progenitors also took on a myofibroblast fate that led to kidney fibrosis and kidney failure. Cell marker studies further confirmed that these myofibroblasts expressed pericyte and resident fibroblast markers, including PDGFR*β*, CSPG4, desmin, GLI1, and NT5E. Notably, increased interstitial collagen deposition associated with loss of *Zeb2* in FOXD1^+^ stromal progenitors was accompanied by increased expression of activated SMAD1/5/8, SMAD2/3, SMAD4, and AXIN2. Thus, our study identifies a key role of ZEB2 in maintaining the cell fate of FOXD1^+^ stromal progenitors during kidney development, whereas loss of ZEB2 leads to differentiation of FOXD1^+^ stromal progenitors into myofibroblasts and kidney fibrosis.

## Graphical abstract

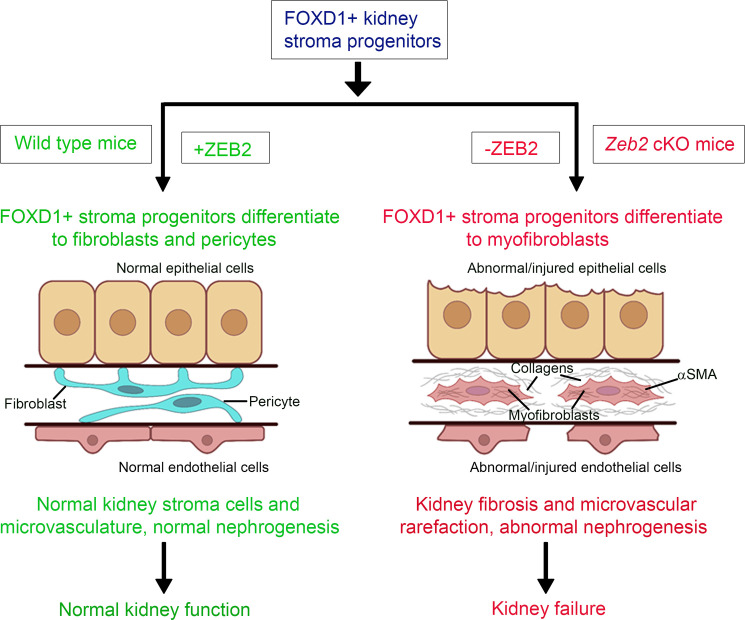

## Introduction

Kidney stromal cells are extracellular matrix–associated support cells in kidney interstitium that are neither tubular epithelial cells nor vascular endothelial cells. These cells are mainly derived from forkhead box D1–positive (FOXD1^+^) kidney stromal progenitor cells that are essential for normal kidney development (1, 2). As kidneys mature, FOXD1^+^ stromal progenitors give rise to interstitium-resident fibroblasts and pericytes that support the kidney vasculature and renal tubules and also cooperate with nephron progenitor cells that give rise to the functional nephron (3).

FOXD1^+^ cell–derived kidney stromal cells, including pericytes and resident fibroblasts, are also the primary precursors of collagen-producing myofibroblasts during kidney fibrosis (4), a major cause of chronic kidney disease (CKD) and kidney failure affecting approximately 13% of the population worldwide (5–7). Upon kidney injury, pericytes proliferate, detach, and migrate from peritubular capillaries, and transdifferentiate into myofibroblasts that produce extracellular matrix proteins such as type 1 collagen in the kidney interstitial space, leading to fibrotic scar formation (4, 8–12). The detachment and migration of pericytes from peritubular capillaries cause destabilization of the capillary wall and loss of endothelial cells, resulting in capillary rarefaction with chronic renal ischemia and hypoxia and eventually nephron disruption, CKD progression, and kidney failure (13, 14). Previous studies have shown that pericytes and resident fibroblasts can be detected with cell-specific markers in the kidney, such as platelet-derived growth factor receptor β (PDGFRβ), chondroitin sulphate proteoglycan 4 (CSPG4, also known as nerve/glial antigen 2 or NG2), desmin, NT5E (also known as CD73 or ecto-5′-nucleotidase [5′NT]), and GLI family zinc finger 1 (GLI1) (4, 11, 15). However, the signals controlling the differentiation of FOXD1^+^ stromal progenitors into pericytes and resident fibroblasts rather than myofibroblasts during kidney development are not known (3, 16).

Zinc finger E-box-binding homeobox 2 or ZEB2 (also known as SIP1 or ZFHX1B) is a member of the ZFH1 family of 2-handed zinc finger/homeodomain transcription factors that promotes epithelial-mesenchymal transition (EMT) (17–19). ZEB2 interacts with activated SMADs and functions as a DNA-binding transcriptional repressor to regulate the TGF-β/BMP signaling pathway (20, 21). TGF-β/SMAD signaling has a critical role in kidney fibrosis. SMAD2 and SMAD3 are activated in the fibrotic kidney in patients and CKD animal models, and *Smad3* deficiency attenuates kidney fibrosis (22–25). *ZEB2* loss-of-function heterozygous mutations in humans cause Mowat-Wilson syndrome (OMIM #235730), a congenital disorder characterized by intellectual disability, craniofacial abnormalities, and Hirschsprung disease (26–30). Over 50% of Mowat-Wilson syndrome patients with *ZEB2* mutations are reported to have renal and genitourinary abnormalities (31–33). However, the pathological features of the kidney defects have not been examined and defined.

Previous studies using cell-specific *Zeb2* conditional knockout (*Zeb2*-cKO) mice have revealed that *Zeb2* plays an essential role in cell fate determination and differentiation of various cell types during development (19, 34–37). We have previously shown that *Zeb2* is crucial for nephron progenitor differentiation during kidney development (38). Deletion of *Zeb2* in nephron progenitors leads to congenital atubular glomeruli and glomerulocystic kidney disease due to abnormal glomerulotubular junction formation in mice (38). Interestingly, our study also showed that ZEB2 is predominantly expressed in the stromal progenitors during kidney development (38). However, the role that ZEB2 plays in stromal progenitor differentiation during kidney development is unknown.

In this study, we tested the hypothesis that ZEB2 is a key protein controlling kidney stromal progenitor cell fate and differentiation. We show that ZEB2 is expressed in FOXD1^+^ stromal progenitors. In the absence of ZEB2, FOXD1^+^ stromal progenitors fail to differentiate into mature stromal cells such as pericytes and resident fibroblasts supporting normal kidney development and instead become myofibroblasts, leading to hypoplastic kidney, kidney fibrosis, and kidney failure.

## Results

### ZEB2 is expressed in the kidney stromal progenitor cells in developing mouse kidney.

**Figure 1:**
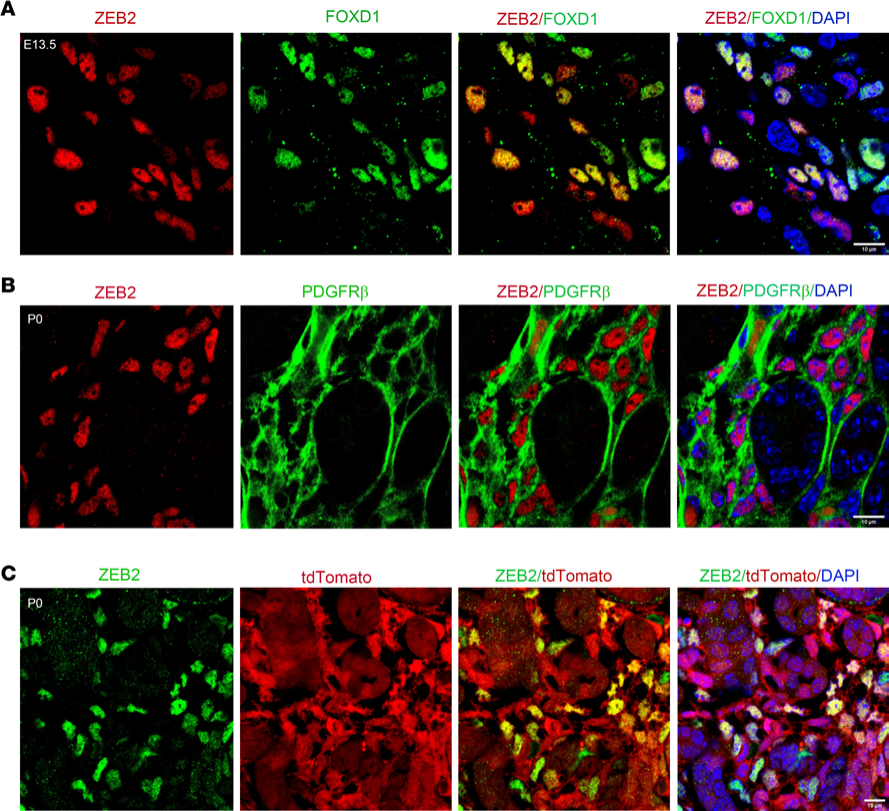
ZEB2 is expressed in kidney stromal cells in the developing mouse kidney. (**A**) Colocalization of ZEB2 (red) and FOXD1 (green, stromal progenitor marker) in E13.5 developing mouse kidney. ZEB2 (red) is also colocalized with DAPI (blue) that binds DNA in cell nuclei. (**B**) Costaining of ZEB2 (red, in cell nuclei) and PDGFRβ (green, on cell plasma membrane) expression in the newborn mouse kidney. (**C**) Colocalization of ZEB2 (green) with tdTomato by using *Foxd1*-*Cre*^+^;tdTomato mice. DAPI (blue) labels cell nuclei. Scale bars: 10 μm (**A**–**C**).

We have previously shown that loss of ZEB2 in nephron progenitors leads to glomerulocystic kidney disease without renal fibrosis (38). Interestingly, the expression pattern of ZEB2 in the kidney suggests that ZEB2 is predominantly expressed in the kidney stromal progenitor cells, although not exclusively (38). To confirm stromal progenitor cell expression of ZEB2 in the developing mouse kidney, we performed double immunostaining of ZEB2 and FOXD1, a marker for kidney stromal progenitors (1). In the embryonic day 13.5 (E13.5) mouse kidney, we found that ZEB2 is colocalized with FOXD1 in the kidney cortex region (Figure 1A). To determine whether ZEB2 is also expressed in stromal cells derived from *Foxd1^+^* stromal progenitors, we examined PDGFRβ, a marker expressed by *Foxd1^+^* cell–derived stromal cells (4). We found that ZEB2 is expressed in the nuclei of PDGFRβ^+^ stromal cells in the newborn mouse kidney (Figure 1B). By lineage tracing in *Foxd1*-*Cre*;tdTomato mice, we also found that ZEB2 is expressed in the medullary stromal cells that are tdTomato^+^, suggesting that these cells derive from *Foxd1^+^* stromal progenitors (Figure 1C). FOXD1 is only expressed in the cortical region and cortical FOXD1-expressing cells are reported to be a multipotent self-renewing progenitor population that gives rise to cortical and medullary interstitial cells, mesangial cells, and pericytes throughout kidney organogenesis (39). FOXD1 expression is downregulated as cortical stroma progenitor cells differentiate into medullary cells (39). These data suggest that ZEB2 is expressed in both FOXD1^+^ stromal progenitors and FOXD1^+^ cell–derived stromal cells in developing mouse kidneys, albeit ZEB2 appears to be more widely expressed in cells other than FOXD1^+^ stromal progenitors.

### Loss of Zeb2 in FOXD1^+^ stromal progenitors leads to kidney failure and early mortality.

**Figure 2:**
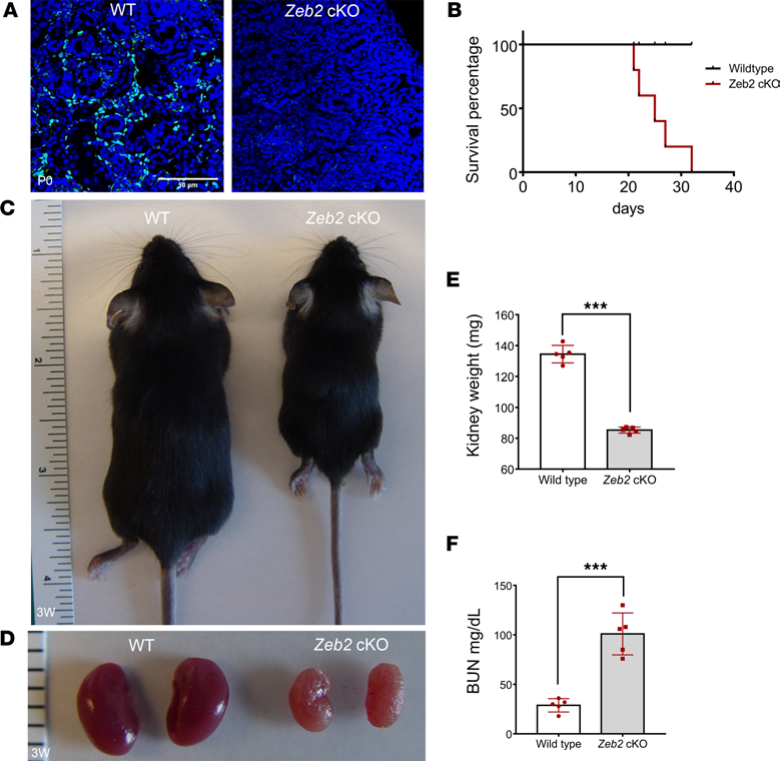
Loss of ZEB2 in FOXD1*^+^* stromal progenitors leads to kidney failure and early mortality. (**A**) Expression of ZEB2 protein in *Zeb2*-cKO mice and wild-type (WT) littermate controls (P0). Compared with WT littermates, there is little ZEB2 (green) expression in the *Zeb2*-cKO mouse kidney. Scale bar: 50 μm. (**B**) *Zeb2*-cKO mice (red line) have shorter life span compared with WT littermate controls (black line). Log-rank test was used for survival analysis: *P* < 0.05. (**C**) Smaller body size in a 3-week-old *Zeb2*-cKO mouse compared with a WT littermate control mouse. (**D**) Three-week-old *Zeb2*-cKO mice have paler and smaller kidneys compared with WT littermate controls. (**E**) The kidney weight of 3-week-old *Zeb2*-cKO mice is significantly reduced compared with their WT littermate controls. (**F**) Elevated blood urea nitrogen (BUN) in 3-week-old *Zeb2*-cKO mice compared with WT littermate controls. Number of animals analyzed: *n* = 5 per group. Data are expressed as mean ± SEM. ***P < 0.001 by 2-tailed, unpaired Student’s *t* test (**E** and **F**).

To understand the in vivo function of *Zeb2* in FOXD1*^+^* stromal progenitors, we generated *Zeb2*-cKO mice (*Zeb2^fl/fl^*;*Foxd1-Cre^+^*) by crossing *Zeb2*-floxed mice with *Foxd1-Cre^+^* mice (4, 39, 40). We confirmed the loss of ZEB2 protein as well *Zeb2* mRNA using immunostaining and in situ hybridization, respectively, in the *Zeb2*-cKO mouse kidney (Figure 2A and Supplemental Figure 1; supplemental material available online with this article; https://doi.org/10.1172/jci.insight.158418DS1). *Zeb2-*cKO mice were viable at birth but subsequently started to die at around 4–5 weeks of age, while control littermates had a normal life span (Figure 2B). Moreover, the body size of 3-week-old *Zeb2*-cKO mice was smaller and they had hunched posture as compared with their wild-type littermates (Figure 2C). Further examination of the *Zeb2*-cKO mice revealed a significant reduction in kidney weight as compared with wild-type littermate controls and the kidneys of 3-week-old *Zeb2*-cKO mice had stiff texture (Figure 2, D and E, and Table 1), suggesting postnatal kidney hypoplasia and fibrotic lesions. To determine whether the kidney function is impaired in the *Zeb2*-cKO mice, we measured the blood urea nitrogen (BUN) and found that the 3-week-old *Zeb2*-cKO mice had nearly 4-fold increased BUN levels (100.6 ± 18.9 mg/dL in *Zeb2*-cKO mice) as compared with their wild-type littermates (26 ± 7.2 mg/dL), indicating impaired kidney function and kidney failure (Figure 2F and Table 1). Interestingly, we did not observe proteinuria in *Zeb2*-cKO mice (Supplemental Figure 2A), suggesting that the glomerular filtration barrier was preserved in these mice. As FOXD1 is also active late in development in podocytes (41, 42), we analyzed the expression of nephrin and podocin, 2 podocyte markers in *Zeb2*-cKO mouse kidneys. We did not observe any change in expression of nephrin and podocin in the *Zeb2*-cKO mouse kidneys compared with the wild-type littermates, suggesting no podocyte defects in the *Zeb2*-cKO mice (Supplemental Figure 2, B and C).

### Loss of Zeb2 in FOXD1^+^ stromal progenitors leads to kidney fibrosis.

**Figure 3:**
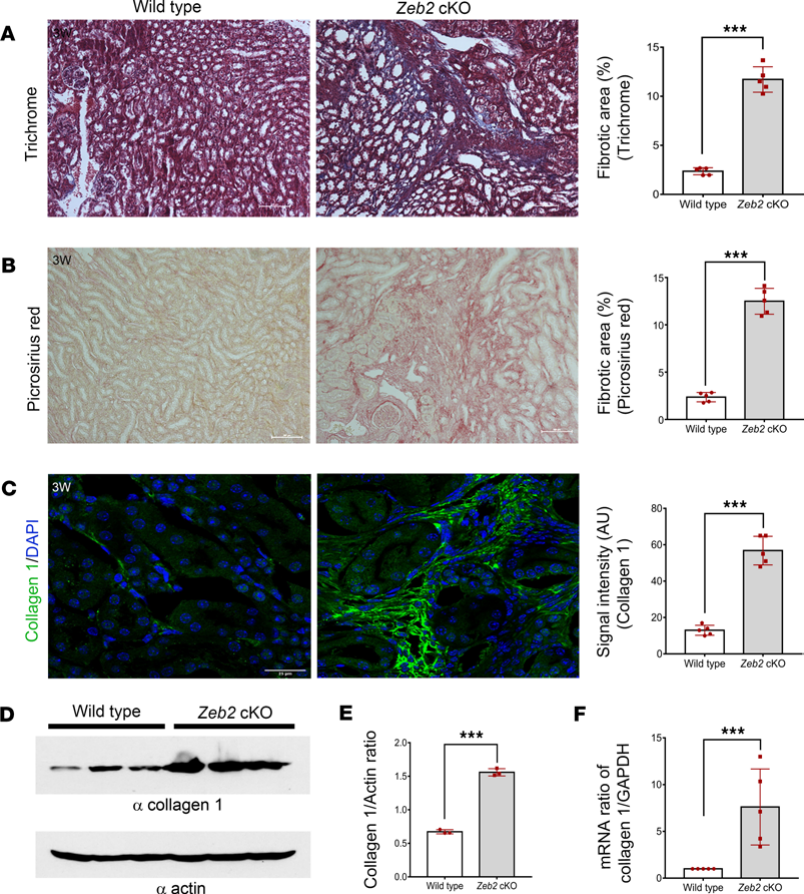
Loss of ZEB2 in FOXD1*^+^* stromal progenitors leads to kidney fibrosis. (**A**) Masson’s trichrome and (**B**) picrosirius red staining and quantification indicate significant increase in matrix deposition (as evident by blue staining in **A** and pink staining in **B**) in 3-week-old *Zeb2*-cKO mouse kidney compared with their wild-type littermate controls. (**C**) Immunostaining and (**D**) Western blot analysis of collagen I in the whole kidney lysate show an increase in collagen I expression in 3-week-old *Zeb2*-cKO mouse kidney as compared with their wild-type littermate controls. Actin was used as a loading control in **D**. (**E**) The intensity ratio and quantification of collagen I versus actin from Western blot in **D**. (**F**) TaqMan real-time quantitative RT-PCR analysis of collagen I mRNA normalized to *Gapdh*. AU, arbitrary unit. Number of animals analyzed: *n* = 5 per group. For Western blot, *n* = 3 per group. Scale bars: 100 μm (**A** and **B**) and 25 μm (**C**). Data are expressed as mean ± SEM. A 2-tailed, unpaired Student’s *t* test was used for statistical significance. ***P < 0.001.

In our previous study, we found that metanephric mesenchyme-specific *Zeb2*-cKO (*Zeb2^fl/fl^;Six2-Cre^+^*) mice develop glomerulocystic disease without renal fibrosis (38). Interestingly, we did not observe any glomerular cystic phenotype in stromal cell–specific *Zeb2*-cKO mice (*Zeb2^fl/fl^;Foxd1-Cre^+^*) (Supplemental Figure 3), suggesting a different underlying cause for kidney failure and early mortality in these mice (Figure 2, B and F). By Mason’s trichrome and picrosirius red staining histopathological analysis, we found significantly more collagen fiber deposition in the kidneys of stromal progenitor–specific *Zeb2*-cKO mice as compared with their wild-type littermate controls (Figure 3, A and B). As type 1 collagen is the major collagen fiber in kidney fibrosis, we examined the expression of collagen type 1, α-1 (COL1A1) by immunostaining and Western blotting analyses. We found that COL1A1 expression was significantly increased in *Zeb2*-cKO mice compared with their littermate controls (Figure 3, C–E). These results were also confirmed by quantitative real-time RT-PCR of *Col1A1* mRNA extracted from kidneys at the same age (Figure 3F). Taken together, these findings indicate that loss of *Zeb2* in FOXD1^+^ stromal progenitors causes kidney fibrosis and kidney failure.

**Figure 4:**
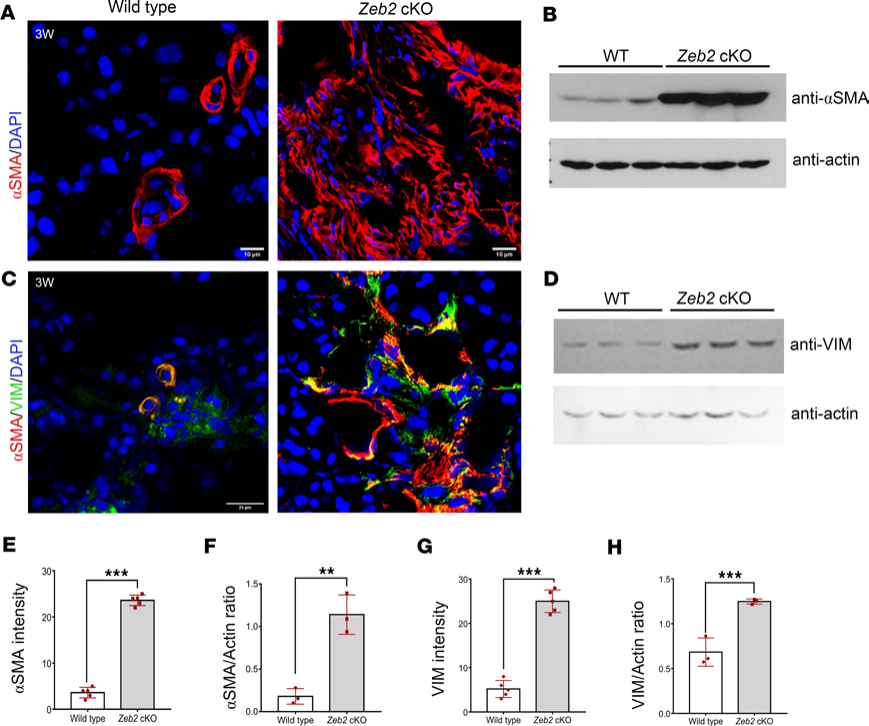
Increased expression of α-SMA and vimentin in 3-week-old *Zeb2*-cKO mouse kidney compared with their wild-type littermate controls. (**A**) Immunofluorescent staining of α-SMA. (**B**) Western blot analysis of α-SMA in whole kidney lysates; actin was used as a loading control. (**C**) Double immunofluorescent staining of α-SMA (red) and vimentin (VIM, green). (**D**) Western blot analysis of VIM in whole kidney lysates; actin was used as a loading control. (**E**) Quantification of α-SMA^+^ intensity in **A**. (**F**) Intensity ratio of α-SMA versus actin. (**G**) Quantification of VIM^+^ intensity in **C**. (**H**) Intensity ratio of VIM versus actin. Number of animals analyzed: *n* = 5 per group. For Western blot, *n* = 3 per group. Scale bars: 10 μm (**A**) and 25 μm (**C**). Data are expressed as mean ± SEM. A 2-tailed, unpaired Student’s *t* test was used for statistical significance. **P < 0.01, ***P < 0.001.

Myofibroblasts with smooth muscle cell–like contractile properties are the major collagen matrix–producing cells in kidney fibrosis (9, 43). Myofibroblasts are characterized by the expression of α smooth muscle actin (α-SMA) that is the major actin isoform in contractile smooth muscle cells (43). To determine whether there is an increase in myofibroblast cells in the kidney of *Zeb2*-cKO mice, we performed immunofluorescent staining using an anti–α-SMA antibody. We found that 3-week-old *Zeb2*-cKO mice had significantly increased α-SMA expression in the kidney as compared with wild-type littermates (Figure 4, A and E). Increased α-SMA expression in *Zeb2*-cKO mice was further confirmed by Western blotting analysis (Figure 4, B and F). By analyzing vimentin, another established myofibroblast marker (44), we confirmed that α-SMA^+^vimentin^+^ myofibroblasts were significantly increased in *Zeb2*-cKO mouse kidneys compared with wild-type controls (Figure 4, C and G). Increased vimentin expression in *Zeb2*-cKO mice was further confirmed by Western blotting analysis (Figure 4, D and H). These data suggest that loss of *Zeb2* in FOXD1^+^ stromal progenitors leads to expansion of the α-SMA^+^vimentin^+^ myofibroblasts that contribute to the kidney fibrosis phenotype.

### The abnormal myofibroblasts are derived from FOXD1^+^ stromal progenitors and express fibroblast and pericyte markers in Zeb2-cKO fibrotic kidney.

**Figure 5:**
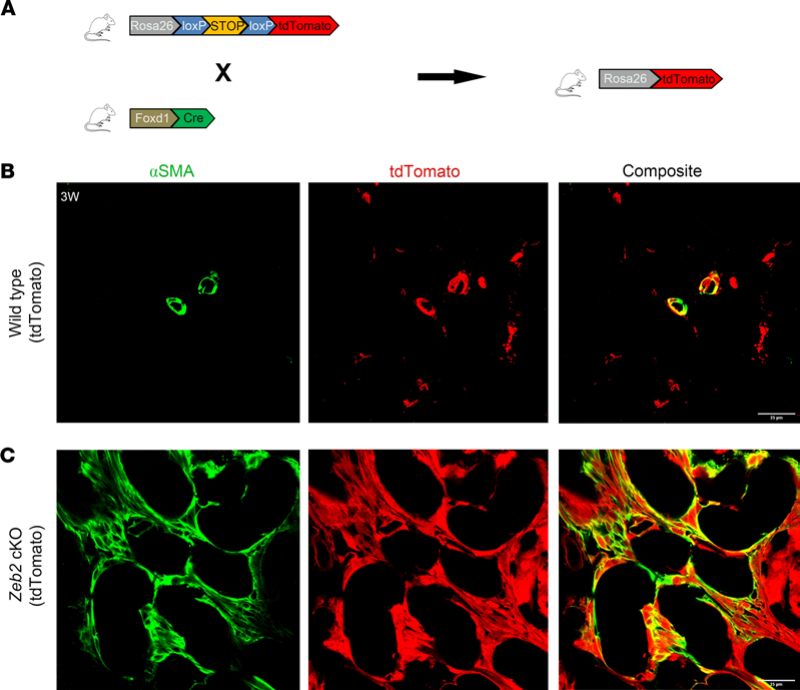
FOXD1^+^ stromal progenitors transdifferentiate into myofibroblasts in the absence of ZEB2. (**A**) Breeding scheme for genetic cell fate mapping of *Foxd1^+^* stromal progenitors using Rosa26-tdTomato mice. (**B**) In the wild-type mouse kidney, tdTomato (red) and α-SMA (green) are only expressed in vascular smooth muscle cells. (**C**) In *Zeb2*-cKO mouse kidney, the α-SMA^+^ myofibroblasts (green) also express tdTomato (red), indicating that α-SMA^+^ myofibroblasts are derived from *Foxd1^+^* stromal progenitors. Number of animals analyzed, *n* = 5 per group. Scale bars: 25 μm (**B** and **C**).

Previous studies showed that FOXD1*^+^* cell–derived stromal cells are the major progenitors for myofibroblasts in kidney fibrosis (4). To determine whether the increased myofibroblasts in the kidney of *Zeb2*-cKO mice are also derived from FOXD1*^+^* stromal progenitors, we performed lineage tracing/fate mapping of FOXD1*^+^* stromal progenitors using the Ai9 tdTomato reporter mice, as previously reported (45, 46). We generated *Zeb2^+/+^;Foxd1-Cre^+^;tdTomato^+^* control mice and *Zeb2^fl/fl^;Foxd1-Cre^+^;tdTomato^+^* cKO mice, in which the daughter cells derived from FOXD1*^+^* stromal progenitors are permanently labeled with red tdTomato reporter signals (Figure 5A). We performed immunofluorescent staining to label α-SMA, the marker for normal smooth muscle cells or pathological myofibroblasts. We observed that tdTomato and α-SMA were predominantly colocalized in vascular smooth muscle cells (VSMCs) in the wild-type mouse kidney (Figure 5B). In *Zeb2*-cKO mice, however, there was a significant increase in abnormal myofibroblasts that expressed both α-SMA and tdTomato (Figure 5C), indicating that these α-SMA^+^ myofibroblasts were derived from FOXD1*^+^* stromal progenitors in the absence of ZEB2.

**Figure 6:**
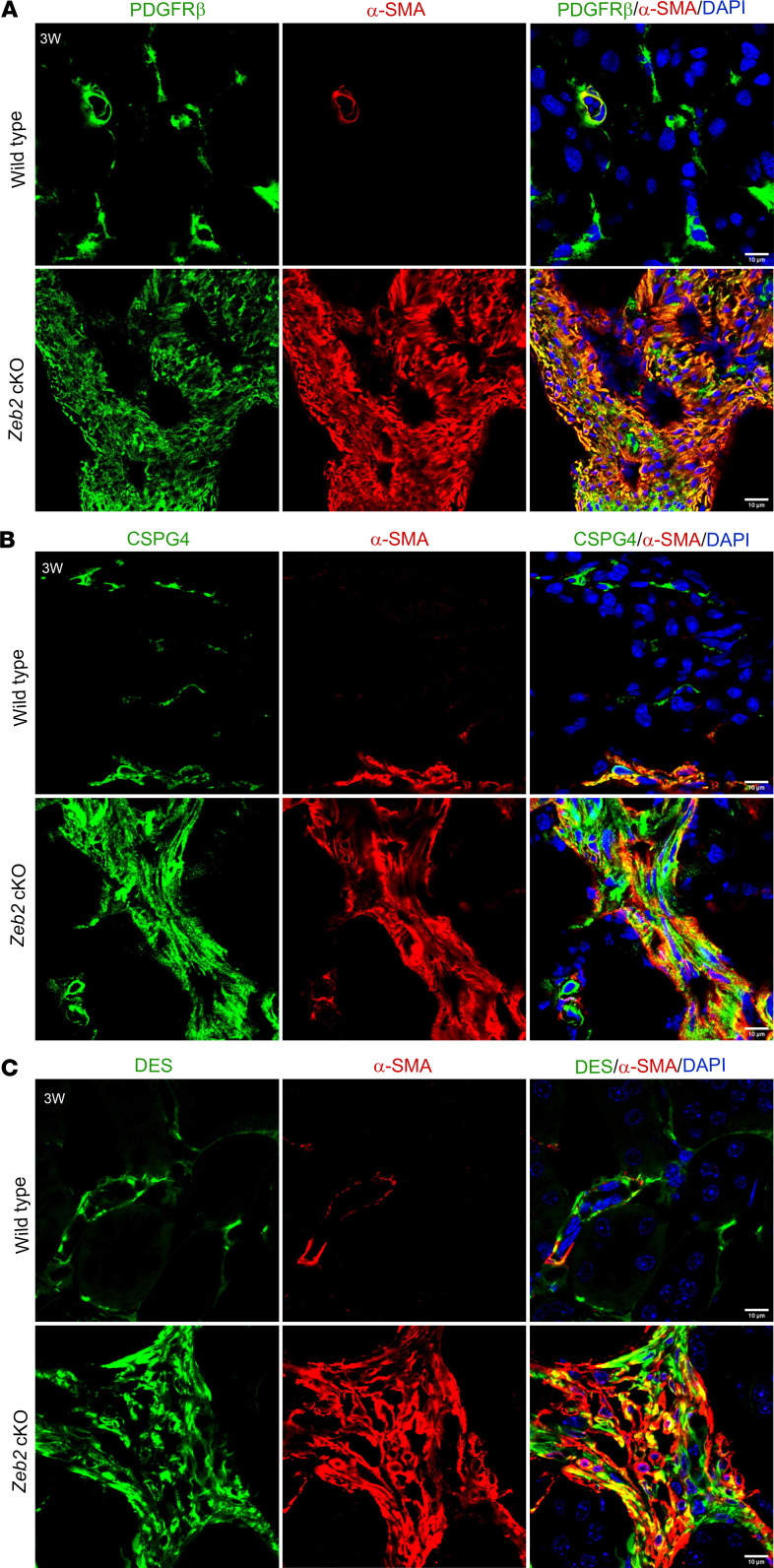
The abnormal myofibroblasts express fibroblast and pericyte markers PDGFRβ, CSPG4, and desmin in *Zeb2*-cKO mouse kidney. Colocalization staining of α-SMA (red) and fibroblast/pericyte markers (green) PDGFRβ (**A**), CSPG4 (**B**), and desmin (DES) (**C**) in the 3-week-old *Zeb2*-cKO mouse kidney as compared with wild-type littermate controls. In wild-type mice, α-SMA is expressed only in vascular smooth muscle cells and not expressed by pericytes/fibroblasts. *n* = 5 per group. Scale bars: 10 μm (**A**–**C**).

**Figure 7:**
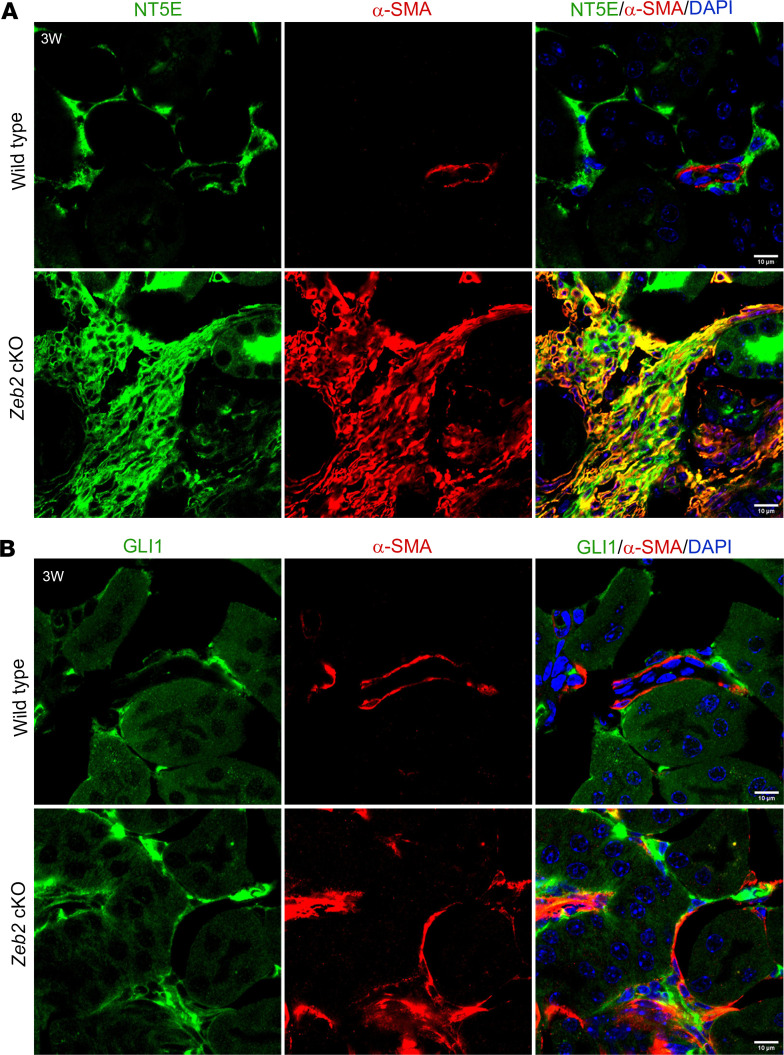
The abnormal myofibroblasts express fibroblast and pericyte markers NT5E and GLI1 in *Zeb2*-cKO mouse kidney. Colocalization staining of α-SMA (red) and fibroblast/pericyte markers (green) NT5E (**A**) and GLI1 (**B**) in the 3-week-old *Zeb2*-cKO mouse kidney as compared with wild-type littermate controls. In wild-type mice, α-SMA is expressed only in vascular smooth muscle cells and not expressed by pericytes/fibroblasts. *n* = 5 per group. Scale bars: 10 μm (**A** and **B**).

Experimental studies on animal models of kidney fibrosis have also demonstrated that FOXD1*^+^* cell–derived α-SMA^+^ myofibroblasts express fibroblast and pericyte markers (4, 11, 12). To determine whether α-SMA^+^ abnormal myofibroblasts in *Zeb2*-cKO mice also express these markers, we performed double immunostaining using α-SMA and fibroblast and pericyte marker antibodies. Indeed, we found that α-SMA^+^ abnormal myofibroblasts also partially expressed fibroblast and pericyte markers, including PDGFRβ, CSPG4, desmin, NT5E, and GLI1 (Supplemental Table 1), as these different markers were all aberrantly colocalized with high α-SMA expression in the *Zeb2*-cKO mouse fibrotic kidney (Figure 6, A–C, and Figure 7, A and B). Normal fibroblasts and pericytes in the wild-type mouse kidney also express these markers at a lower level without increased expression of α-SMA (Figure 6, A–C, and Figure 7, A and B). These data suggest that loss of ZEB2 leads to pericyte/fibroblast-to-myofibroblast cell fate switch and *Zeb2* controls the differentiation of a subset of FOXD1^+^ stromal progenitors into pericytes and fibroblasts during kidney development.

### FOXD1^+^ stromal progenitors differentiate abnormally in the absence of ZEB2.

**Figure 8:**
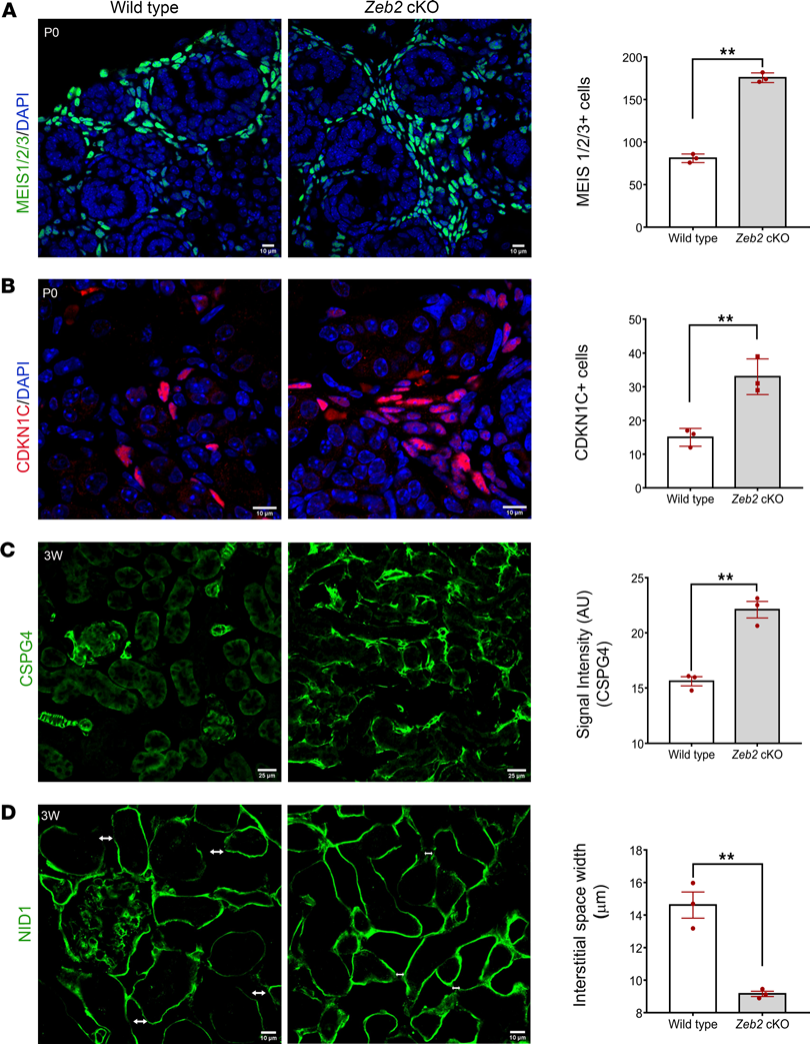
FOXD1^+^ stromal progenitors differentiate abnormally in *Zeb2*-cKO mice. (**A** and **B**) Increased expression of stromal cell markers MEIS1/2/3 and CDKN1C in newborn *Zeb2*-cKO mice as compared with their wild-type littermate controls. (**C**) Immunostaining of stromal cell marker CSPG4 in 3-week-old (3W) *Zeb2*-cKO mouse kidney shows abnormal expression of CSPG4 in the kidney cortical region. (**D**) Basement membrane marker nidogen-1 staining shows a significant reduction in the interstitial space width (↔) in *Zeb2*-cKO mouse kidney as compared with their wild-type littermate controls. *n* = 3 per group. Scale bars: 10 μm (**A**, **B**, and **D**) and 25 μm (**C**). Data are expressed as mean ± SEM. A 2-tailed, unpaired Student’s *t* test was used for statistical significance. **P < 0.01.

To determine whether the FOXD1^+^ stromal progenitor cells differentiate properly during early kidney development in *Zeb2*-cKO kidneys, we analyzed the expression of known stromal cell differentiation markers, such as MEIS1/2/3 (47, 48), CDKN1C, and CSPG4 (NG2) (Supplemental Table 1) (39, 49). By immunofluorescent staining and confocal microscopy analyses, we found that the kidney of newborn *Zeb2*-cKO mice had significantly more MEIS1/2/3^+^ stromal cells in the kidney cortical and medullary region and the distribution of MEIS1/2/3^+^ stromal cells was also altered as compared with wild-type littermate controls (Figure 8A). The kidney medullary region of newborn *Zeb2*-cKO mice also had significantly more CDKN1C^+^ stromal cells as compared with wild-type controls (Figure 8B). Furthermore, in 3-week-old *Zeb2*-cKO mice, cortical stromal cells also had abnormally high CSPG4 (NG2) expression that is a stromal cell marker, normally expressed in the VSMCs and mesangial cells in the kidney (Figure 8C). These data suggest that FOXD1^+^ stromal progenitors did not differentiate normally in the absence of ZEB2. As stromal cells such as residential fibroblasts reside in the kidney interstitial space (39), we checked whether there is any abnormality in the interstitial space due to abnormal stromal cell differentiation in the *Zeb2*-cKO mouse kidney. By analyzing interstitial space width using the basement membrane marker nidogen-1 (50), we found that the kidneys of 3-week-old *Zeb2*-cKO mice had an abnormally narrow interstitial space as compared with their wild-type littermate controls (Figure 8D). Taken together, these data suggest that myofibroblasts in *Zeb2*-cKO mouse kidney are derived from abnormally developed FOXD1^+^ stromal progenitor cells and not from mature renal fibroblasts or pericytes.

### Zeb2-cKO mice develop vascular rarefaction and abnormal nephrogenesis.

**Figure 9:**
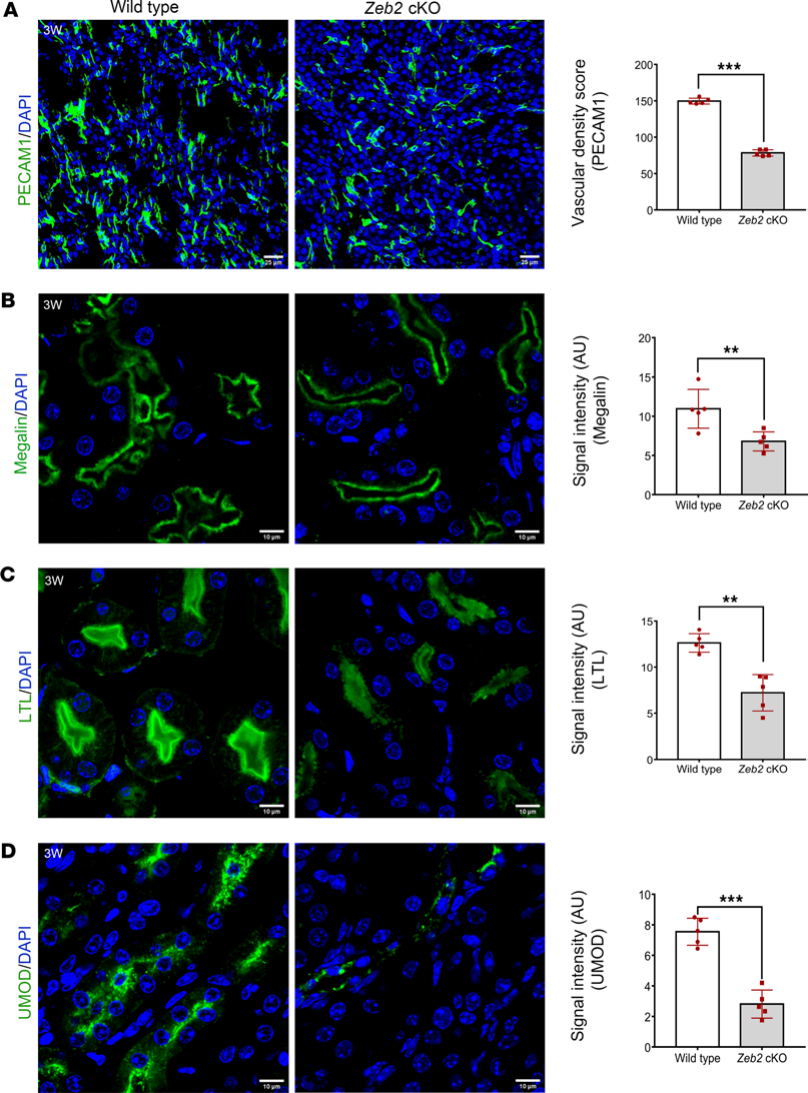
*Zeb2*-cKO mice develop vascular rarefaction and abnormal kidney tubule formation. (**A**) A significant decrease in endothelial cells revealed by its marker PECAM1 in 3-week-old (3W) *Zeb2*-cKO mice compared with wild-type littermate controls. (**B** and **C**) Staining for the proximal tubular markers megalin and *Lotus tetragonolobus* lectin (LTL) shows abnormal proximal tubule structure and reduced signal intensity in *Zeb2*-cKO mice compared with wild-type littermate controls. (**D**) UMOD staining shows abnormal distal tubule development and reduced signal intensity in *Zeb2*-cKO mice as compared with wild-type littermate controls. AU, arbitrary unit. *n* = 5 per group. Scale bars: 25 μm (**A**) and 10 μm (**B**–**D**). Data are expressed as mean ± SEM. A 2-tailed, unpaired Student’s *t* test was used for statistical significance. **P < 0.01, ***P < 0.001.

Kidney stromal cells are important in supporting microvascular development and stability, and loss of stromal cells can lead to vascular rarefaction and abnormal nephron development (13, 14). To determine whether abnormal stromal cell development in *Zeb2*-cKO mice also causes vascular rarefaction and abnormal nephrogenesis, we performed immunostaining to examine the microvascular integrity in the kidney using the endothelial marker PECAM1. We found that *Zeb2*-cKO kidneys had significantly low vascular density as compared with their wild-type littermate controls (Figure 9A), suggesting a microvascular rarefaction. Using megalin and *Lotus tetragonolobus* lectin (LTL), 2 proximal tubule markers, we found an abnormal proximal tubule structure and reduced megalin and LTL signal intensity in *Zeb2*-cKO kidneys compared with wild-type littermate controls, suggesting abnormal proximal tubule development in the *Zeb2*-cKO mice (Figure 9, B and C). By uromodulin (UMOD) immunostaining, we found that development of the loops of Henle and distal tubules were also impaired in *Zeb2*-cKO mouse kidneys as compared with wild-type controls (Figure 9D).

**Figure 10:**
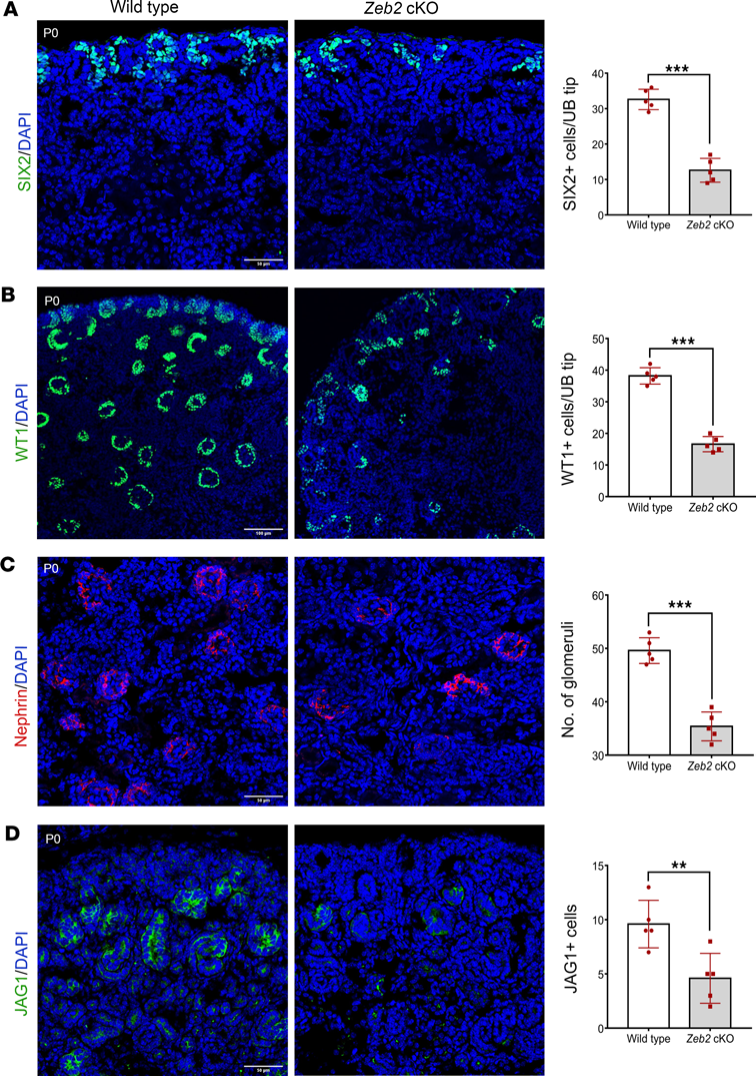
Newborn *Zeb2-*cKO mice have abnormal nephrogenesis. (**A**) Newborn *Zeb2*-cKO kidneys have fewer SIX2^+^ nephron progenitors as compared with wild-type littermate controls. UB, ureteric bud. (**B**–**D**) *Zeb2*-cKO mouse kidneys also had significantly fewer nephron numbers that was confirmed by WT1, nephrin, and Jagged1 (JAG1) immunostaining. *n* = 5 per group. Scale bars: 50 μm (**A**, **C**, and **D**) and 100 μm (**B**). Data are expressed as mean ± SEM. A 2-tailed, unpaired Student’s *t* test was used for statistical significance. **P < 0.01, ***P < 0.001.

*Foxd1^+^* stromal progenitors also regulate the differentiation of nephron progenitors during kidney development (48, 51–55). As *Foxd1^+^* stromal progenitors did not differentiate properly in *Zeb2*-cKO mice, we examined nephrogenesis by analyzing several markers that are important for nephron progenitors and normal nephron formation. Our data showed that newborn *Zeb2*-cKO kidneys had fewer SIX2^+^ nephron progenitors as compared with wild-type littermate controls (Figure 10A). *Zeb2* homozygous newborn cKO kidneys also had significantly fewer nephrons, as demonstrated by immunostaining of Wilms tumor 1 (WT1, a marker for metanephric mesenchyme and glomeruli), nephrin (a marker for podocytes), and Jagged1 (a marker for developing proximal tubules) (Figure 10, B–D). Taken together, these findings support the conclusion that ZEB2 is crucial for proper differentiation of FOXD1*^+^* stromal progenitors that are required for renal microvasculature integrity and nephron development.

### Loss of Zeb2 in Foxd1^+^ stromal progenitors leads to enhanced SMAD and WNT signaling.

**Figure 11:**
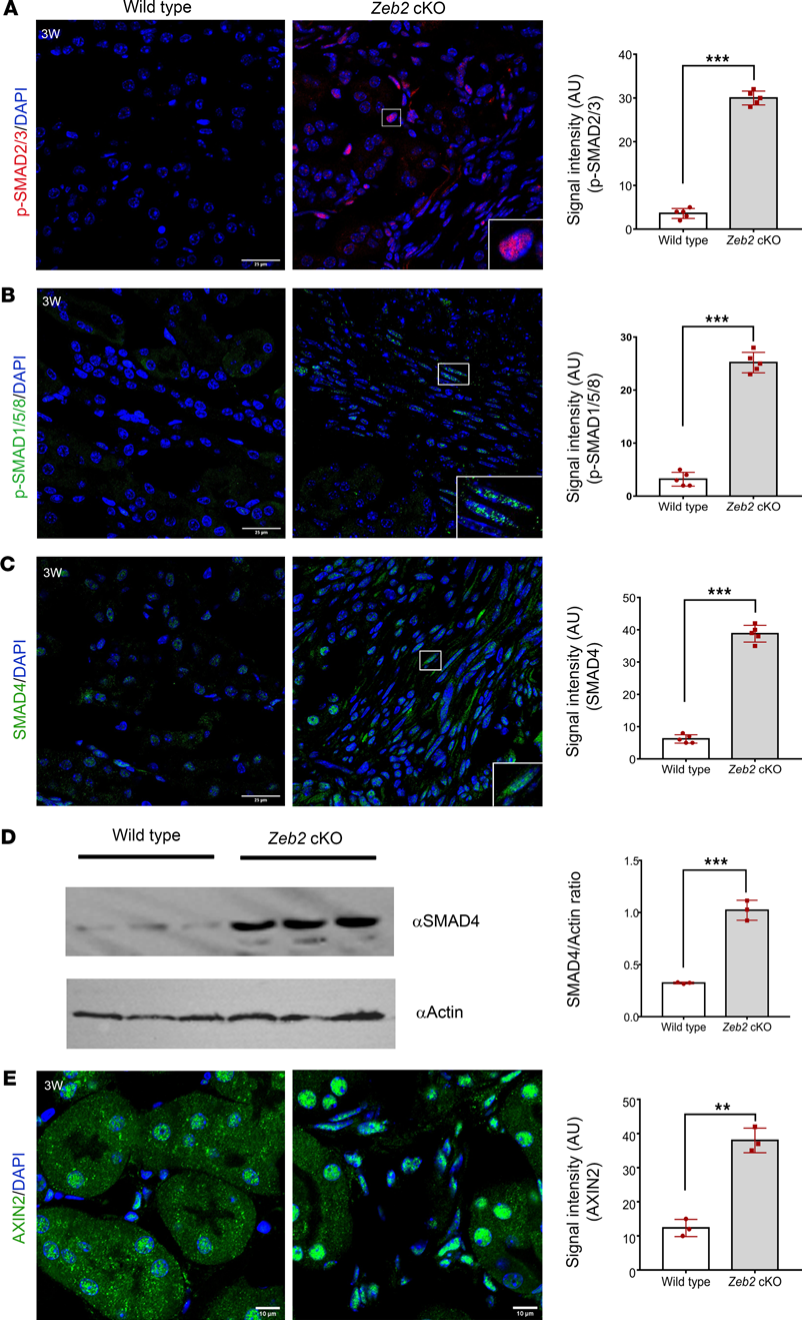
Loss of ZEB2 in FOXD1*^+^* stromal progenitors leads to enhanced SMAD and WNT signaling. (**A**–**C**) Immunofluorescent staining of p-SMAD2/3, p-SMAD1/5/8, and SMAD4 in 3-week-old (3W) *Zeb2*-cKO and their wild-type littermate kidneys together with quantifications of p-SMAD2/3^+^, p-SMAD1/5/8^+^, and SMAD4^+^ cell staining intensity. (**D**) Western blot analysis of SMAD4 in whole kidney lysates; actin was used as a loading control. Intensity ratio of SMAD4 versus actin was statistically analyzed. (**E**) Immunofluorescent staining of AXIN2 in 3-week-old *Zeb2*-cKO and their wild-type littermate kidneys. AU, arbitrary unit. Boxed regions show enlarged images. *n* = 5 per group. For Western blot, *n* = 3 per group. Scale bars: 25 μm (**A**–**C**) and 10 μm (**E**). Data are expressed as mean ± SEM. A 2-tailed, unpaired Student’s *t* test was used for statistical significance. **P < 0.01, ***P < 0.001.

ZEB2 is also called SMAD-interacting protein 1 (SIP1). It contains a SMAD-binding domain and forms protein complexes with activated R-SMADs, including SMAD1, SMAD2, SMAD3, SMAD5, and SMAD8 (56). SMAD signaling also plays a critical role in kidney fibrosis (23). As *Zeb2*-cKO mice developed kidney fibrosis, we asked whether there was any change in SMAD signaling. We used anti–phospho-R-SMAD antibodies to label the activated forms of R-SMAD and found that the expression of p-SMAD2/3 and p-SMAD1/5/8 was significantly increased in the *Zeb2*-cKO mouse kidney compared with their wild-type controls (Figure 11, A and B). Interestingly, we found that SMAD4, a co-SMAD, was also upregulated in the *Zeb2*-cKO mouse kidney by immunostaining (Figure 11C) and was confirmed by Western blotting (Figure 11D).

In addition to SMAD signaling, ZEB2 also modulates WNT signaling during organogenesis (57). Interestingly, published studies have shown that activation of WNT signaling promotes kidney fibrosis in both mouse and human kidneys (58–60). To determine whether WNT signaling is also activated in *Zeb2*-cKO kidneys, we analyzed the expression of AXIN2 protein, an important component of the WNT signaling pathway (61). We found that expression of AXIN2 was upregulated in *Zeb2*-cKO mouse kidneys as compared with wild-type controls (Figure 11E). Taken together, these data suggest that loss of *Zeb2* in FOXD1*^+^* stromal progenitors leads to enhanced activity of both the SMAD and WNT signaling pathways that may contribute to the kidney fibrosis phenotype in the *Zeb2*-cKO mice.

## Discussion

**Figure 12:**
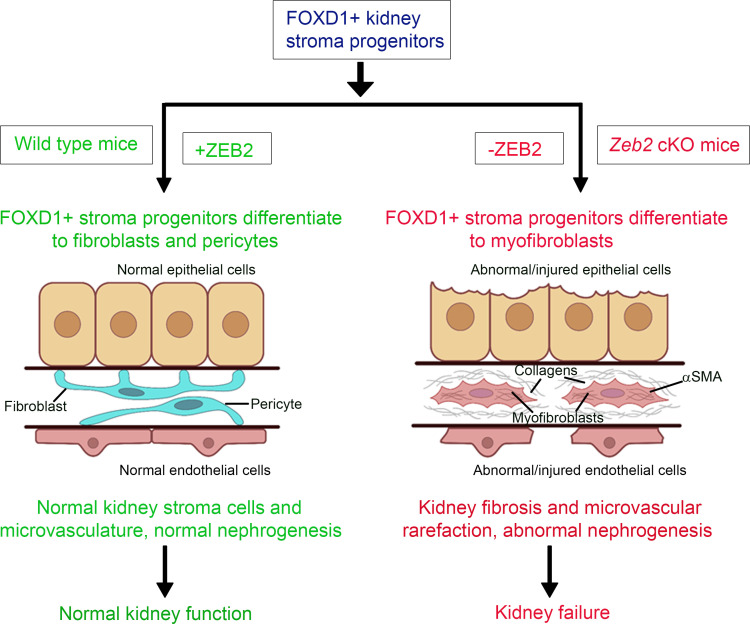
Proposed model. ZEB2 controls kidney stromal progenitor cell differentiation and inhibits abnormal myofibroblast expansion and kidney fibrosis.

In the present study, we show that the transcription factor ZEB2 is crucial for FOXD1*^+^* stromal progenitor development and differentiation in the developing mouse kidney. ZEB2 protein is expressed in the cortical and medullary stromal cells and is colocalized with FOXD1 in cortical stromal progenitors in the E13.5 developing embryonic kidney. Loss of ZEB2 in FOXD1*^+^* stromal progenitors causes the kidney stromal progenitor cells to differentiate into myofibroblasts instead of pericytes and fibroblasts. This is associated with endothelial cell loss, microvascular rarefaction, impaired nephrogenesis, interstitial fibrosis, and kidney failure (Figure 12). Our studies, thus, identified ZEB2 as a factor in maintaining normal FOXD1^+^ stromal progenitor cell differentiation during kidney development.

Previous reports have shown that stromal cells derived from the *Foxd1^+^* stromal progenitors are the major precursors for myofibroblasts during kidney fibrosis and these abnormal myofibroblasts have increased expression of fibroblast and pericyte marker genes during kidney injury (4, 62). This is consistent with our findings, as myofibroblasts in *Zeb2*-cKO mice are derived from FOXD1*^+^* stromal progenitors and express fibroblast and pericyte markers such as PDGFRB, CSPG4, desmin, NT5E, and GLI1. However, the published studies were often conducted in adult mice or rats with surgery-induced kidney injuries such as ischemia reperfusion injury (IRI) or unilateral ureter obstruction (UUO) (4, 63–65). In contrast, genetic deletion of *Zeb2* in FOXD1*^+^* stromal progenitors spontaneously causes them to differentiate into myofibroblasts instead of normal stromal cells such as resident fibroblasts and pericytes. These data demonstrate that genetic loss of a key gene such as *Zeb2* in FOXD1*^+^* stromal progenitors is sufficient to drive spontaneous myofibroblast formation in the absence of induced kidney injury and thus provides a non–surgically induced animal model for kidney fibrosis. Our studies may also have translational potential, as a recent publication on the myofibroblast origins in human kidney fibrosis confirms pericytes and fibroblasts as the main cellular sources of scar-forming myofibroblasts during human kidney fibrosis (60).

Little is known about how the FOXD1*^+^* stromal progenitors give rise to mature stromal cells in the developing kidney. Previously, it has been reported that pericytes express markers such as PDGFRβ and CSPG4 as well as the myofibroblast marker α-SMA up to postnatal day 12. As the kidney matures, however, pericytes lose their α-SMA expression (12). After UUO-induced kidney injury in 12-day-old mice, pericytes retain both CSPG4 and α-SMA expression and transdifferentiate into myofibroblasts (12). More recently, it has shown that partial UUO injury in newborn mice also leads to changes in the FOXD1*^+^* stromal progenitor cell fate and FOXD1*^+^* stromal progenitors transdifferentiate into myofibroblasts after kidney injury (66). Published reports suggest that FOXD1*^+^* stromal progenitors may differentiate into myofibroblasts, depending on different cellular and molecular signals during kidney development (12, 66). Our studies establish that ZEB2 is one such key factor that regulates these cellular and molecular signals in the kidney stromal progenitor cells. In the absence of ZEB2, FOXD1*^+^* stromal progenitor cells take on a myofibroblast fate during kidney development. These findings are in line with previous reports (60, 66). Additional experiments are needed to identify other factors and the key cellular and molecular pathways that are downstream of ZEB2 and essential for FOXD1*^+^* cell–derived stromal cell differentiation in the developing kidney.

FOXD1*^+^* stromal progenitors differentiate into pericytes during kidney development (4, 39). Pericytes play an important role in the stability and integrity of the microvasculature through the synthesis of basement membrane and factors that control vascular tone and angiogenesis (13, 15, 67). Loss of pericytes can lead to microvascular rarefaction and tubular atrophy and injury in the kidney (13, 14). Interestingly, we also found that *Zeb2*-cKO mice have vascular rarefaction and abnormal tubule formation. This was likely due to the lack of pericytes, which differentiated into myofibroblasts in the absence of ZEB2 in stromal cell progenitors. Our data suggest that ZEB2 is crucial in maintaining the FOXD1*^+^* stromal progenitor cell fate, including pericytes, during kidney development.

The molecular mechanism of how ZEB2 regulates lineage specification of FOXD1*^+^* stromal progenitors during kidney development is also partially unraveled by this study. ZEB2 is a SMAD-binding protein in the TGF-β/SMAD/BMP pathway (56) and studies in different organ systems demonstrated that ZEB2 is a negative regulator of SMAD signaling during development (20, 37). Previously it was shown that SMADs are expressed during mouse kidney development (68). Based on the observed patterns of expression, it has been speculated that individual or combinations of SMADs may play a specific role in cell-fate determination during kidney development (68). In addition, SMAD signaling also plays a critical role in kidney fibrosis (23). SMAD2 and SMAD3 are activated in the fibrotic kidney in patients and animal models with CKD, and *Smad3*-KO mice are protected from kidney fibrosis after UUO injury (22, 24, 25). Given the important role of TGF-β/SMAD signaling in kidney fibrosis and early lineage specification (23, 69) and recent data on the role of SMAD signaling in kidney interstitial cell proliferation (70), we hypothesize that loss of ZEB2 may lead to activation of SMAD signaling in stromal progenitors and thereby affect their lineage specification in the developing kidney. Indeed, we found upregulation of several activated R-SMADs (e.g., p-SMAD3 and p-SMAD1/5/8) and the co-SMAD (i.e., SMAD4) in the *Zeb2*-cKO fibrotic kidney. This supports our conclusion that loss of ZEB2 causes misdirected differentiation of kidney stromal progenitors into myofibroblasts instead of mature stromal cells (such as pericytes and fibroblasts) in the developing kidney, leading to kidney fibrosis through enhanced SMAD signaling. In addition, our data suggest that enhanced WNT signaling may also contribute to the abnormal differentiation of kidney stromal progenitors into myofibroblasts in the *Zeb2*-cKO kidney. Interestingly, a WNT signaling negative regulator, NKD2, was recently identified as a myofibroblast-specific therapeutic target in human kidney fibrosis (60). Further studies are needed to better understand the molecular mechanism of ZEB2 in modulating the WNT signaling pathway during kidney development and kidney fibrosis.

FOXD1*^+^* stromal progenitors also regulate the renewal and differentiation of nephron progenitors during kidney development (48, 51–55). Interestingly, a previous study showed that the *Zeb2* promoter region contains binding sites for FOXD1, which inhibits the expression of *Zeb2* and decorin (*Dcn*), another gene that antagonizes SMAD signaling during stromal cell development and is required for nephron progenitor differentiation (52). Another genetic study also showed that haploinsufficiency of *Zeb2* in transgenic rats leads to retardation of nephrogenesis (71). We also found that nephrogenesis is impaired in *Zeb2*-cKO mice. Abnormal development of kidney stromal cells in *Zeb2-*cKO mice causes pericyte and endothelial cell loss and microvascular rarefaction, which may impair nephron development or causes postdevelopmental tubular atrophy. Alternatively, it is possible that ZEB2 may have a more direct, as yet undefined, function in supporting nephrogenesis. Rather than simply replacement of atrophic tubules with extracellular matrix, our studies also show that the development of interstitial fibrosis is an active process resulting from myofibroblast proliferation as a result of ZEB2 deficiency in FOXD1^+^ stromal progenitors. Additional studies may help us to understand the crosstalk between ZEB2 and FOXD1 during kidney development and in the regulation of nephron progenitor differentiation.

In summary, our study demonstrates an essential role of ZEB2 in FOXD1*^+^* stromal progenitor differentiation and development in the kidney. In the absence of ZEB2*,* FOXD1*^+^* stromal progenitors take on a myofibroblast fate, which results in abnormal expansion of myofibroblasts, collagen deposition, kidney fibrosis, as well as microvascular rarefaction and abnormal nephrogenesis. In the presence of ZEB2, FOXD1^+^ stromal progenitors differentiate into mature stromal cells, including pericytes and perivascular fibroblasts, which promote microvascular and nephron development and normal kidney function (Figure 12). Thus, our study identifies *ZEB2* as a gene that is essential in maintaining FOXD1^+^ stromal progenitor cell differentiation during kidney development. These findings have implications for understanding the underlying developmental abnormalities affecting the kidney in Mowat-Wilson syndrome patients with *ZEB2* loss-of-function mutations. Future studies may contribute to the growing body of knowledge about the role of stromal progenitor cells, pericytes, resident fibroblasts, and myofibroblasts in acquired kidney diseases associated with interstitial fibrosis.

## Methods

### Animals.

*Zeb2*-floxed mice and the genotyping protocol were described previously (40). *Foxd1-Cre^+^* (stock 012463) and Rosa26tdTomato (stock 007909) mice were purchased from the Jackson Laboratory and genotyped according to the protocol as provided by the Jackson Laboratory. Eight-week-old *Zeb2^fl/+^*;*Foxd1-Cre^+^* mice were bred with *Zeb2^fl/fl^* mice to generate *Zeb2^fl/fl^*;*Foxd1-Cre^+^* homozygous cKO mice. All mice had free access to drinking water and a standard rodent diet ad libitum. Animals of both sexes in a mixed C57BL/6 × 129 genetic background were used in this study. Littermate wild-type mice were used as controls.

### Tissue preparation and histology.

The kidneys of 3-week-old *Zeb2*-cKO mice and wild-type littermate controls were dissected and fixed in 4% paraformaldehyde (PFA) or 10% neutral buffered formalin overnight and processed for paraffin embedding following standard protocols. Serial kidney sections were cut and stained using a Periodic Acid Schiff’s (PAS) Stain Kit (24200, Polysciences, Inc.), Picrosirus Red Stain Kit (24901, Polysciences, Inc.), and Masson’s Trichrome Stain Kit (25088, Polysciences, Inc.) according to the manufacturer’s instructions. Slides were examined with an Olympus upright light microscope and photographed using an Olympus DP72 digital camera.

### Blood serum and urine analysis.

Blood was collected from 3-week-old *Zeb2*-cKO mice and wild-type littermate controls. Briefly, the mice were anesthetized and euthanized using CO_2_ and whole blood was collected by heart puncture. Blood was then incubated at room temperature for 30 minutes followed by centrifugation at 2100*g* to collect the serum that was then stored at –80°C. Serum BUN was measured using a Catalyst Dx Chemistry Analyzer (IDEXX Laboratories). For analysis of proteinuria, spot urine was collected from 3-week-old *Zeb2*-cKO mice and wild-type littermate controls and stored at –80°C. Urine protein was examined by sodium dodecyl sulphate–polyacrylamide gel electrophoresis (SDS-PAGE) followed by Coomassie blue staining using an albumin standard.

### Immunofluorescent staining and confocal microscopy.

For immunofluorescent staining, mouse kidneys at different developmental stages were harvested. For E13.5 kidneys, the embryos were harvested from timed pregnant females. Kidney tissues were dissected and snap frozen or fixed in 4% PFA overnight followed by incubation in 30% sucrose at 4°C overnight. Kidney tissues were then embedded in optimal cutting temperature (OCT) compound (Tissue-Tek, Sakura Finetek) and sectioned at 8–10 μm thickness at –20°C using a Microm HM550 cryostat (Thermo Fisher Scientific). Frozen kidney sections were permeabilized with PBS containing 0.1% Triton X-100 for 10 minutes and blocked in 5% serum blocking buffer for 1 hour at room temperature. Primary antibodies were incubated at 4°C overnight followed by secondary antibodies incubated at room temperature for 1 hour. The following primary antibodies (Supplemental Table 2) were used: ZEB2 (HPA003456, Sigma-Aldrich; 1:100 dilution), FOXD1 (sc-47585, Santa Cruz Biotechnology; 1:100 dilution), desmin (MA5-13259, Thermo Fisher Scientific; 1:20 dilution), PDGFRβ (sc-432, Santa Cruz Biotechnology [1:100 dilution] and APB5, Thermo Fisher Scientific [1:100 dilution]), CSPG4 (AB5320, Sigma-Aldrich; 1:200 dilution), PECAM1 (550274, BD Biosciences; 1:100 dilution), α-SMA (A254, Sigma-Aldrich [1:200 dilution] and ab5694, Abcam [1:100 dilution]), vimentin (sc-6260, Santa Cruz Biotechnology; 1:50 dilution), MEIS1/2/3 (39096, Active Motif ; 1:200 dilution), CDKN1C (sc-8298, Santa Cruz Biotechnology; 1:50 dilution); nidogen-1 (NBP1-97701, Novus Biologicals; 1:100 dilution), GLI1 (UM870063, OriGene Technologies, Inc.; 1:50 dilution), CD73 (550738, BD Biosciences; 1:100 dilution), SIX2 (11562-1-AP, Proteintech; 1:400 dilution), WT1 (ab89901, Abcam; 1:100 dilution), JAG1 (sc-8303; Santa Cruz Biotechnology; 1:50 dilution), nephrin (AF3159, R&D Systems; 1:50 dilution), LTL (FL-1321, Vector Labs; 1:200 dilution), megalin (sc-16478, Santa Cruz Biotechnology; 1:50 dilution), UMOD (MAB5175, R&D Systems; 1:50 dilution), COL1A1 (ab21286, Abcam; 1:50 dilution), SMAD4 (ABE21, Sigma-Aldrich; 1:400 dilution), p-SMAD3 (ab52903, Abcam; 1:300 dilution), p-SMAD1/5/8 (13820, Cell Signaling Technology; 1:100 dilution), AXIN2 (ab32197, Abcam; 1:100 dilution), and podocin (ab50339, Abcam; 1:500 dilution). A list of cell-specific markers used in this study is shown in Supplemental Table 1. The secondary antibodies were donkey anti–rat IgG FITC (712-095-150, Jackson ImmunoResearch Laboratories; 1:200 dilution), goat anti–mouse IgG2a–Alexa Fluor 594 (A-21135, Thermo Fisher Scientific; 1:200 dilution), goat anti–mouse IgG1–Alexa Fluor 488 (A-21121, Thermo Fisher Scientific; 1:200 dilution), rat anti–mouse IgG2a–FITC (11-4210-80, Thermo Fisher Scientific; 1:20 dilution), goat anti–rabbit IgG–Alexa Fluor 488 (ab150077, Abcam; 1:500 dilution), donkey anti–rabbit IgG–Alexa Fluor 488 (ab150073, Abcam; 1:500 dilution), and donkey anti–goat IgG–Alexa Fluor 594 (ab150132, Abcam; 1:500 dilution). Sections were then stained with DAPI (4′,6′-diamidino-2-phenylindole) and mounted in VECTASHIELD antifade mounting medium (H-1000, Vector Labs). Images were captured using a Zeiss LSM 700 confocal microscope and analyzed with ImageJ software (NIH).

### In situ hybridization assay.

The in situ hybridization (RNAscope) assay was performed according to the manufacturer’s instructions with RNAscope Multiplex Fluorescent Detection Kit V2 (323110, Advanced Cell Diagnostics). Briefly, frozen mouse kidney tissues were fixed in 4% PFA in 1× PBS at 4°C for 15 minutes and rinsed twice in 1× PBS. The slides were then dehydrated in 50%, 70%, and 100% ethanol for 5 minutes per incubation. After that, kidney tissues were incubated in hydrogen peroxide for 10 minutes at room temperature, rinsed in water, and then incubated in RNAscope protease IV for 30 minutes at room temperature, followed by 2 washes in 1× PBS. The tissues were then incubated with a *Zeb2* probe (RNAscope Probe Mm-Zeb2, catalog 436391, Advanced Cell Diagnostics) for 2 hours at 40°C, followed by 2 washes in 1× PBS. The probe signal was amplified by incubating with RNAscope Multiplex FL V2 Amp 1 for 30 minutes at 40°C, and then rinsed in 1× PBS twice. Finally, the signal was developed by incubating with RNAscope Multiplex FL V2 C1, and subsequently with Opal 570 (FP1488001KT, Akoya Biosciences; 1:1000 dilution). After rinsing twice in 1× PBS, the slides were counterstained in DAPI and mounted in Prolong Diamond antifade mounting medium (P36970, Invitrogen). Images were captured using a Zeiss LSM 700 confocal microscope and analyzed with ImageJ software.

### Image quantification.

Quantification of images was performed using ImageJ on random images (*n* = 5 per mouse). Cell counter or particle analysis plugin tools were used to quantify the cell number. For intensity quantification, the percentage of total pixel area was quantified.

### Western blot analysis.

To examine the α-SMA, vimentin, SMAD4, and COL1A1 expression, 3-week-old *Zeb2*-cKO and wild-type littermate mouse kidneys were homogenized in a lysis buffer (1× PBS, 0.5% Triton X-100, 1× protease inhibitor) on ice. Samples were centrifuged at 4°C for 10 minutes and the supernatants were transferred to new tubes and mixed with a protein loading buffer (BP-111R, Boston Bioproducts, Inc.). Kidney lysates were then heated at 95°C for 20 minutes and resolved in 10% SDS-PAGE gels. Proteins were transferred to PVDF membranes and blotted with mouse anti–α-SMA antibody (A2547, Sigma-Aldrich; 1:1000 dilution), anti-vimentin (5741, Cell Signaling Technology; 1:1000 dilution), SMAD4 (ABE21, Sigma-Aldrich; 1:1000 dilution), or anti-COL1A1 antibody (ab21286, Abcam; 1:1000 dilution). Mouse anti–β-actin antibody was used as a loading control (MA5-15739, Thermo Fisher Scientific; 1:1000 dilution). The images were captured with exposure or with an iBright FL1500 Imaging System (A44241, Thermo Fisher Scientific). The intensity of protein signal bands was measured and quantified using NIH ImageJ analysis software.

### TaqMan real-time PCR gene expression analysis.

Kidney samples of 3-week-old *Zeb2*-cKO and wild-type littermate control mice were collected and preserved in RNAprotect Tissue Reagent (76104, QIAGEN) and stored at –80°C. Total RNA was extracted from the kidneys using the RNeasy Micro kit (74004, QIAGEN) according to the manufacturer’s instructions. cDNA was synthesized from total RNA using the Verso cDNA Synthesis Kit (AB-1453, Thermo Fisher Scientific) as per the manufacturer’s recommended protocols. Gene expression was analyzed using the 7500 FAST real-time PCR machine (Applied Biosystems) with mouse *Col1a1* TaqMan probe (Mm00801666-g1, Thermo Fisher Scientific). Relative gene expression data were analyzed by the ΔΔCt method and were normalized to *Gapdh* according to published methods (72).

### Statistics.

All statistical analyses were performed using GraphPad Prism statistical software (version 7.0). A *P* value of less than 0.05 was considered statistically significant. Data from wild-type and *Zeb2*-cKO mice were compared using a 2-tailed, unpaired Student’s *t* test. The log-rank test was used for survival analysis. Data are represented as mean ± SEM. A minimum of 3 mice were used for each analysis, unless stated otherwise.

### Study approval.

All animal experiments were performed according to the protocol approved by the Institutional Animal Care and Use Committee (IACUC) of Boston University Medical Campus (approved IACUC protocol number PROTO201800056).

## Author contributions

SK and WL conceived and designed the experiments. SK, XF, HMR, and RS performed the experiments and acquired the data. SK, XF, DJS, and WL analyzed the data. DJS and WL provided reagents, materials, and analytical tools. SK and WL wrote the manuscript. All authors reviewed and approved the final manuscript.

## Supplementary Material

Supplemental data

## Figures and Tables

**Table 1:**
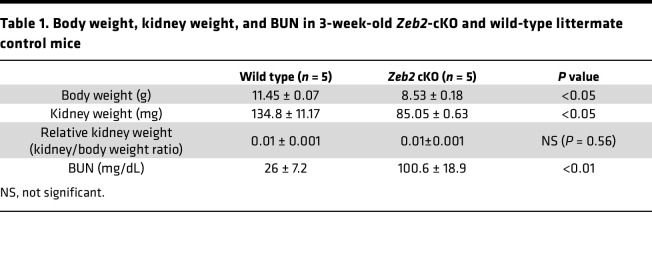
Body weight, kidney weight, and BUN in 3-week-old *Zeb2*-cKO and wild-type littermate control mice

## Version 1 11/29/2022

In-Press Preview

## Version 2 01/10/2023

Electronic publication
