# Supplementary material for: ZEB2 controls kidney stromal progenitor differentiation and inhibits abnormal myofibroblast expansion and kidney fibrosis
Source: JCI Insight. 2023 Jan 10;8(1):e158418. doi: 10.1172/jci.insight.158418 (PMC9870089; doi:10.1172/jci.insight.158418)
Supplement: Supplemental data [file jciinsight-8-158418-s130.pdf]

Supplementary Data

ZEB2 controls kidney stromal progenitor differentiation and inhibits abnormal myofibroblast expansion and kidney fibrosis

Sudhir Kumar, Xueping Fan, Hila Milo Rasouly, Richa Sharma, David J. Salant, Weining Lu*

Supplementary Figures and figure legends

Supplementary Figure 1

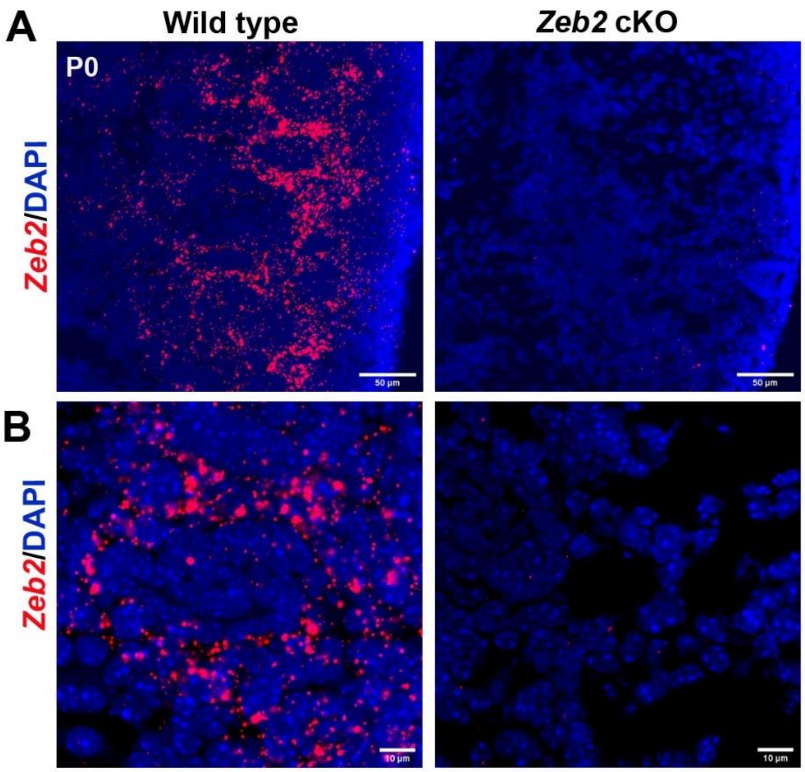

Supplementary Figure 1: Analysis of *Zeb2* expression in *Zeb2* cKO mice by RNAscope in situ hybridization. In wild type mice, *Zeb2* mRNA is expressed in the interstitial cells, but it is absent in *Zeb2* cKO mice. DAPI (blue) labels cell nuclei. Scale bar is 50 for panel **A** and 10 μm for panel **B**.

Supplementary Figure 2

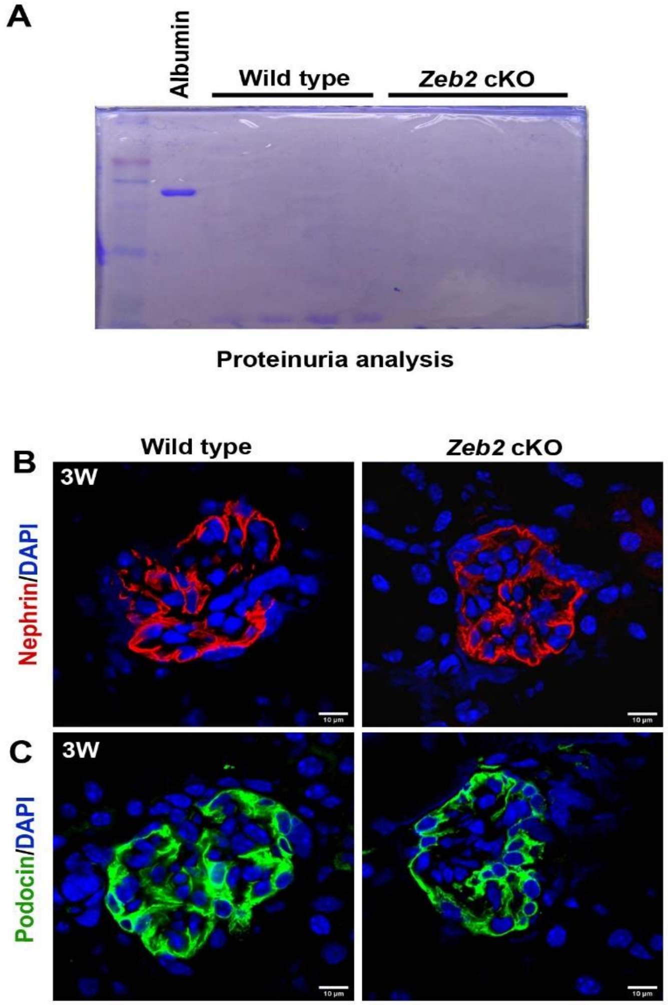

Supplementary Figure 2: No proteinuria and podocyte defects are observed in 3-week-old *Zeb2* cKO mice. (A) *Zeb2* cKO mice did not develop proteinuria as compared to wild type littermate controls. Albuminuria was examined by sodium dodecyl sulphate–polyacrylamide gel electrophoresis (SDS-PAGE) and Coomassie blue staining. N = 3 per group. Albumin used as control. **(B, C)** There was no change in nephrin and podocin expression in *Zeb2* cKO mouse kidneys as compared to wild type mice. Scale bar is 10 µm for panels **B** and **C**.

Supplementary Figure 3

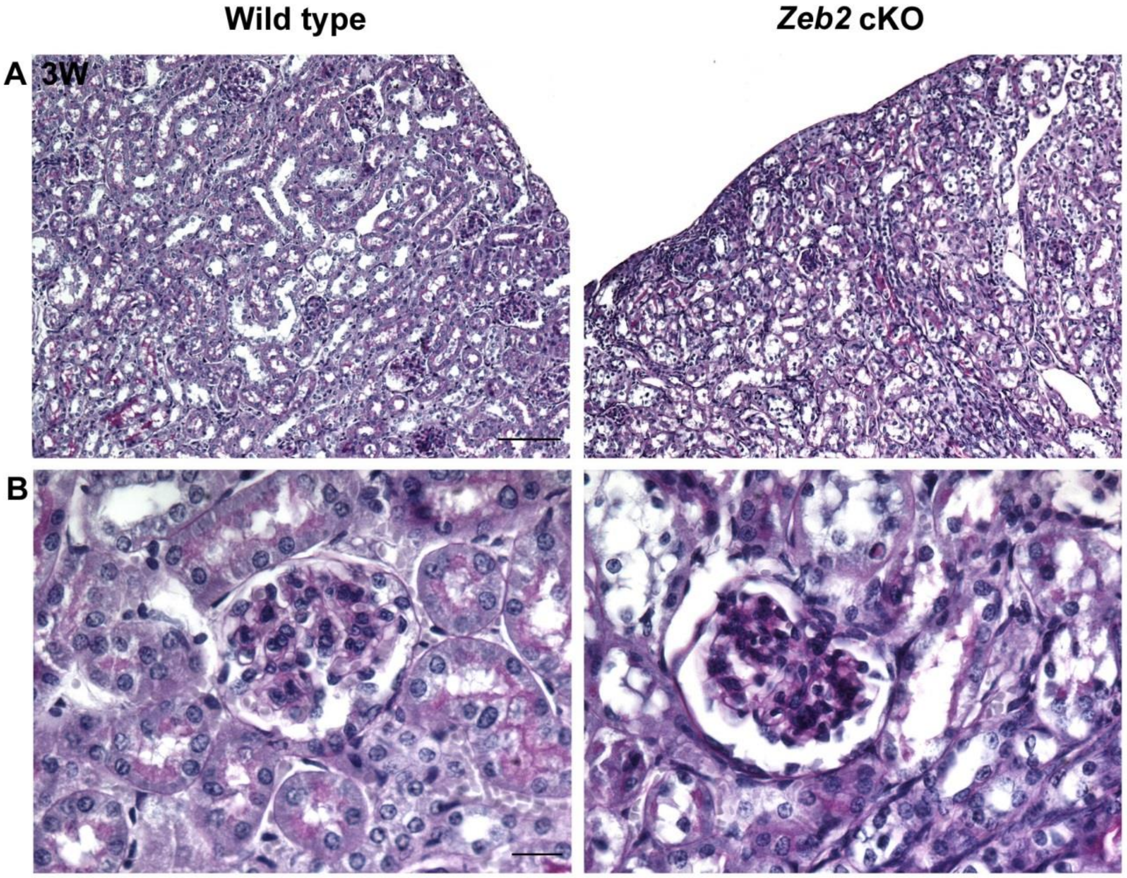

Supplementary Figure 3: Loss of ZEB2 in FOXD1^{+} stromal progenitors did not cause glomerulocystic disease. PAS staining show relative normal glomerular morphology in 3-week-old *Zeb2* cKO kidney as compared to their wild type littermate controls. Scale bar is 100 µm for panel **A** and 20 µm for panel **B**. n = 5 per group.

Supplementary Tables

Supplementary Table 1: Cell specific markers used in this study

Protein Name	Cell specific marker
FOXD1	Kidney stromal progenitor
PDGFRβ	Stromal cell/resident fibroblast and pericyte
α-SMA	Myofibroblast, VSMC
Vimentin	Myofibroblast, VSMC
CSPG4 (NG2)	VSMC and mesangial cell/pericyte
Desmin	VSMC and mesangial cell/pericyte
NT5E/CD73	Mesenchymal cell/Resident fibroblast
GLI1	Mesenchymal cell/pericyte
Meis1/2/3	Kidney stromal cell
CDKN1C	Kidney stromal Cell
Nidogen-1	Basement membrane
SIX2	Nephron progenitor
WT1	Nephron progenitor
JAG1	Developing proximal tubule

VSMC: vascular smooth muscle cells

Supplementary Table 2: Primary antibodies used in this study

Protein	Catalog #	Species	Manufacturer	Application (dilution)
ZEB2	HPA003456	Rabbit	Sigma-Aldrich USA	IF (1:100)
FOXD1	sc-47585	Goat	Santa Cruz Biotechnology USA	IF (1:100)
Desmin	MA5-13259	Mouse	ThermoFisher Scientific USA	IF (1:20)
PDGFRβ	sc-432	Rabbit	Santa Cruz Biotechnology USA	IF (1:100)
PDGFRβ	APB5	Rat	ThermoFisher Scientific USA	IF (1:100)
CSPG4	AB5320	Rabbit	Sigma-Aldrich USA	IF (1:200)
PECAM1	550274	Rat	BD Biosciences USA	IF (1:100)
α-SMA	A2547	Mouse	Sigma-Aldrich USA	IF (1:200), WB (1:1000)
α-SMA	ab5694	Rabbit	Abcam USA	IF (1:100)
Vimentin	sc-6260	Mouse	Santa Cruz Biotechnology USA	IF (1:50), WB (1:1000)
MEIS1/2/3	39096	Mouse	Active Motif USA	IF (1:200)
CDKN1C	sc-8298	Rabbit	Santa Cruz Biotechnology USA	IF (1:50)
Nidogen-1	NBP1-97701	Rat	Novus Biologicals USA	IF (1:100)
GLI1	UM870063	Mouse	OriGene Technologies USA	IF (1:50)
CD73	550738	Rat	BD Biosciences USA	IF (1:100)
SIX2	11562-1-AP	Rabbit	Proteintech USA	IF (1:400)
WT1	ab89901	Rabbit	Abcam USA	IF (1:100)
JAG1	sc-8303	Rabbit	Santa Cruz Biotechnology USA	IF (1:50)
Nephrin	AF3159	Goat	R&D systems USA	IF (1:50)

LTL	FL-1321	Plant	Vector Labs USA	IF (1:200)
Megalin	sc-16478	Goat	Santa Cruz Biotechnology USA	IF (1:50)
UMOD	MAB5175	Rat	R&D systems USA	IF (1:50)
collagen 1	ab21286	Rabbit	Abcam USA	IF (1:50), WB (1:1000)
SMAD4	ABE21	Rabbit	Sigma-Aldrich USA	IF (1:400), WB (1:1000)
P-SMAD3	ab52903	Rabbit	Abcam USA	IF (1:300)
P-SMAD158	13820	Rabbit	Cell Signaling Technology USA	IF (1:100)
β-actin	MA5-15739	Mouse	ThermoFisher Scientific, USA	WB (1:1000)
AXIN2	AB32197	Rabbit	Cell Signaling Technology USA	IF (1:100)
Podocin	Ab50339	Rabbit	Abcam USA	IF (1:500)
